# Evaluation of Fracture Resistance in CAD-CAM Additively Manufactured Occlusal Veneers

**DOI:** 10.3390/polym18172163

**Published:** 2026-09-04

**Authors:** Georgiana Osiceanu, Roxana Diana Vasiliu, Flavia Roxana Bejan, Nicușor Alin Sîrbu, Raluca Faur, Liliana Porojan

**Affiliations:** 1 Department of Dental Prostheses Technology (Dental Technology), Center for Advanced Technologies in Dental Prosthodontics, Faculty of Dental Medicine, “Victor Babes” University of Medicine and Pharmacy Timisoara, Eftimie Murgu Sq. No. 2, 300041 Timisoara, Romania; roxana.vasiliu@umft.ro (R.D.V.); flavia.toma@umft.ro (F.R.B.); sliliana@umft.ro (L.P.); 2 Doctoral School, Faculty of Dental Medicine, “Victor Babes” University of Medicine and Pharmacy Timisoara, Eftimie Murgu Sq. No. 2, 300041 Timisoara, Romania; 3 National Research & Development Institute for Welding and Material Testing—ISIM Timisoara, 30, Blv. Mihai Viteazu, 300222 Timisoara, Romania; asirbu@isim.ro (N.A.S.); rfaur@isim.ro (R.F.); 4 Faculty of Chemical Engineering, Biotechnology and Environmental Protection, Politehnica University of Timisoara, Bld. Vasile Parvan 6, 300223 Timisoara, Romania

**Keywords:** additive dental technology, 3D-printed occlusal veneers, CAD-CAM materials, fracture resistance, compressive strength, mechanical properties

## Abstract

Three-dimensional (3D) printing technology has become more and more popular in restorative dentistry; however, information regarding the mechanical properties of 3D-printed restorative materials remains limited. The aim of this study was to evaluate the behavior under compressive loading until fracture of occlusal veneers fabricated from two types of 3D-printed resin composites, Saremco Print Crowntec A2 and Voco V-Print C&B Temp A2, intended for permanent and temporary clinical restorations, respectively. The study design involved scanning a first upper premolar typodont tooth, previously prepared to receive an occlusal veneer restoration, followed by the computer-aided design of the occlusal veneers and resin dies and 3D printing, resulting in 20 samples. The cemented restorations were subjected to mechanical testing using a fracture-resistance test at a speed of 5 mm/min, applied until failure. The recorded failure forces ranged between 571 and 970 Newton (N), values that are comparable to physiological masticatory forces. The absorbed energy was calculated as the area under the force–displacement curve using the trapezoidal integration method. The mean energy at failure was 0.301 Joule (J) (Voco) and 0.244 Joule (J) (Saremco), with Voco demonstrating greater toughness. In terms of fracture pattern classification, the 3D-printed resin with a lower filler content presented a more catastrophic failure mode compared with the material with a higher filler content. Fractographic analysis revealed characteristic fracture patterns and failure-specific features. Higher predictability and greater fracture strength were observed for the low-filled material, as indicated by the Weibull analysis.

## 1. Introduction

Dental restorations, as well as natural dental tissues, face numerous challenges in the oral cavity with respect to mechanical stability. Tooth wear is one of these challenges, a problem with significant implications for oral health, as it can lead to functional issues such as vertical dimension decrease, pulpal disease and esthetic compromise [1]. Dietary habits, such as a high intake of fruit beverages and carbonated drinks, are important factors in the etiology of erosive tooth wear because they can reduce the strength of dental hard tissues and increase their vulnerability to mechanical wear [2].

Nowadays, conventional treatment approaches involve the rehabilitation of severely worn teeth with full-coverage crown restorations. However, preparing the teeth for full-coverage crowns involves significant sacrifice of tooth structure, which may lead to pulpal injury and compromise tooth vitality [3]. To overcome these drawbacks and adopt a minimally invasive approach, occlusal veneers (OV) have gained popularity as a reliable method to re-establish functional balance, occlusal integrity and patient satisfaction [4]. The preference for tooth-preserving treatment is also consistent with current European recommendations, which promote conservative restorative strategies, while other restorative procedures should be postponed whenever possible [5]. Also, clinically speaking, minimally invasive treatment is regarded as a key concept in biomimetic dentistry, highlighting the conservation of intact dental tissues in order to maximize the long-term success of restorative procedures [6].

The design of occlusal veneers is considered to play an important role in their long-term performance. Several occlusal veneer designs have been proposed in the literature, including straight-beveled margins, butt-joint preparations, circumferential margins, proximal slots, rounded shoulder margins, chamfer preparations, and various combinations of occlusal and axial surface coverage [7]. Other studies have described preparations such as hollow chamfers (in the occlusal third) and have suggested modified designs, including deep chamfer preparations, as more conservative alternatives for occlusal veneer preparations [8]. However, the existence of an enamel layer at the preparation finish line may significantly affect the mechanical behavior of the restoration [9].

Evolving from conventional manufacturing methods, contemporary restorative dentistry is based on computer-aided technologies for the production of dental restorations, either using Computer-Aided Design and subtractive Computer-Aided Manufacturing CAD/CAM) or additive manufacturing (3D printing). In subtractive manufacturing, the restoration is fabricated by shaping the material from a prefabricated block using computer numerical control (CNC) milling [10]. Although widely used, subtractive manufacturing has several limitations, including material waste and wear of the milling burs, which lead to reduced service life and decreased machining accuracy for complex restorations, as the milling outcome depends on the motion and other characteristics of the cutting instruments [11].

In contrast, the additive manufacturing process consists of the progressive deposition and light-curing of monomeric material in successive layers by means of a computer-guided energy source, which allows the accurate reproduction of complex dental anatomy [12].

These innovations have expanded the possibilities for restoring severely worn dentition, especially when restorative space is restricted [13]. However, there are still insufficient data on the mechanical strength of 3D-printed materials used in minimally invasive restorative approaches. Some studies comparing milled and 3D-printed restorations have shown that 3D-printed samples demonstrate lower fracture strength than milled restorations, while still maintaining clinically acceptable mechanical performance [14,15]. In addition, the layer-by-layer fabrication process results in anisotropic properties [16]. Nevertheless, several other factors, such as the printing process and material structure, influence the mechanical behavior of 3D-printed materials.

A successful dental restoration is evaluated based on three fundamental criteria: fracture resistance, marginal adaptability, and esthetic appearance [17]. A major disadvantage of occlusal veneers is their risk of fracture. During the mastication process, repeated tensile stresses can gradually propagate radial cracks in ceramic restorations, ultimately resulting in fracture [18]. For long-term clinical performance, restorations should be capable of resisting both compressive and tensile forces. These stresses act simultaneously during chewing and result from the maximum voluntary biting force, which is the greatest force produced by the masticatory muscles [18].

Nevertheless, previous research has shown that additive manufacturing technology, due to its satisfactory mechanical characteristics, is recommended for both temporary and permanent dental crowns, offering an alternative to indirect methods for medium- to long-term use [19]. However, despite clinical success reported in some studies, the lower fracture toughness of these materials can increase the risk of failure and reduce restoration longevity [20].

The materials commonly used for occlusal veneer restorations include zirconia, lithium disilicate (considered the preferred material), and feldspathic ceramic [7]. Even though zirconia has multiple advantages, its main limitation is the more demanding cementation procedure, resulting in a greater risk of debonding [21]. Composite resin materials have long been used for indirect restorations. Due to the continuous development of CAD/CAM technology and the advancements in 3D additive manufacturing, 3D-processed composite resins have become some of the newest materials that can be used for permanent prostheses, gaining great interest from both researchers and clinicians [22].

Two representative 3D-printed restorative materials are Saremco Print Crowntec and Voco V-Print C&B Temp. Saremco Print Crowntec is a methacrylic acid ester-based light-curing flowable polymer that may be used to make 3D-printed permanent crowns, inlays, onlays, veneers, temporary crowns and bridges, and dentures (full or partial), as mentioned by the manufacturer [23]. Its composition comprises 30–50% inorganic filler (0.7 µm particle dimension) [23]. Its recent development and the limited available evidence require further research, as its mechanical properties have not been sufficiently studied [24].

On the basis of their internal structure, provisional materials can be divided into two categories: acrylic resin-based materials, including polymethyl methacrylate (PMMA) and polyethyl or polybutyl methacrylate (PEMA), and composite resin-based materials, which contain dimethacrylate monomers such as urethane dimethacrylate (UDMA) and bisphenol A-glycidyl methacrylate (Bis-GMA) [25]. Voco V-Print C&B Temp is a light-curing resin with a 26% filler loading, indicated for the fabrication of long-term, esthetic temporary restorations like bridges and crowns, which can last up to 12 months, according to the manufacturer’s specifications [26].

Therefore, the relatively recent introduction of these two materials, as well as the limited knowledge about their mechanical performance, the relationship between their mechanical properties and internal structure, and their failure behavior, requires further investigation. The mechanical testing can be complemented by additional investigation methods, such as the examination of the fractured surfaces, in order to provide insight into the mechanisms and characteristics of failure. One method for assessing the characteristics of fractured surfaces, detecting key fracture features, including the fracture origin and better understanding failure mechanisms following mechanical testing is fractographic analysis [27]. Qualitative fractographic analysis is the assessment of fractured surfaces through the study of morphological features which offer clues about the sites of crack initiation and the direction of crack extension, as well as mirror and mist zones, hackle markings, Wallner lines and compression curls [28].

Current evidence regarding the use of 3D-printed materials as a reliable option for this conservative approach remains limited. Although conservative treatment approaches are increasingly adopted, the optimal preparation design and restorative material have yet to be established because each material category presents specific benefits and drawbacks. Moreover, there is still a lack of data supporting the use of 3D-printed materials in these indications, as they have not been studied enough.

Due to the fact that mechanical performance is regarded as a significant factor in the lifespan of restorations, fracture resistance has become one of the most important parameters in the prediction of the clinical effectiveness of indirect restorations. Consequently, more studies are required to assess the mechanical performance of new 3D-printable materials and their suitability for conservative restorative purposes. The null hypotheses of this study were formulated as follows:

**H1.**  *There were no statistically significant differences in fracture resistance between the two 3D-printed resin materials.*

**H2.**  *There are no differences in the fractographic characteristics of the two 3D-printed resin materials.*

**H3.**  *There is no difference in the Weibull probability of failure between the two 3D-printed resin materials.*

## 2. Materials and Methods

In this study, two CAD-CAM additive materials were evaluated: Saremco Print Crowntec (Saremco Dental AG, Rebstein, Switzerland) and Voco V-Print C&B Temp (Voco GmbH, Cuxhaven, Germany), A2. Maxcem Elite Clear (SDS Kerr, Orange, CA, USA) was used as the luting cement. The manufacturers and the compositions of all materials used in the study are listed in Table 1.

An upper premolar typodont tooth, compatible with Kilgore Nissin typodont systems (model M8024), was selected as it provides a standardized experimental model and reduces the variability associated with extracted human teeth, such as differences in tooth morphology, dentin thickness, age, the degree of mineralization and previous restorations [29]. Therefore, this method eliminates the disadvantages associated with using human teeth, which may lead to differences between samples that can influence the accuracy of the study outcomes [29].

The typodont tooth was prepared to receive an occlusal veneer, allowing for a more consistent comparison of the mechanical performance of the tested occlusal veneer materials. The preparation involved a 1 mm occlusal reduction using a round-ended cylindrical diamond bur 881.314.010 Komet Dental (Gebr. Brasseler GmbH & Co. KG, Lemgo, Germany) and a rounded shoulder finish line, followed by finishing with a fine-grit bur 8881.314.010 (Komet Dental (Gebr. Brasseler GmbH & Co. KG, Lemgo, Germany) [7,30].

**Figure 1:**
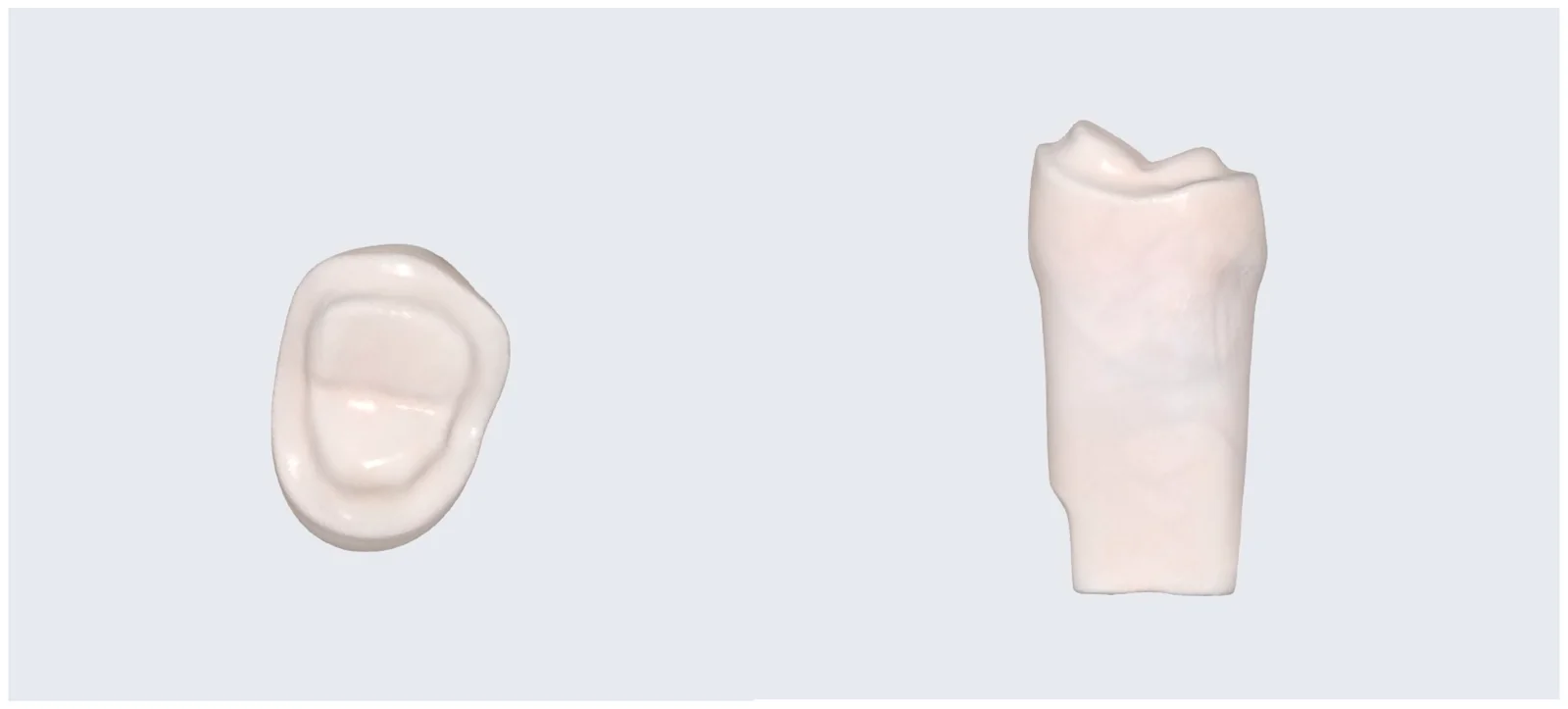
The occlusal veneer preparation was scanned using the Medit i700 scanner (Medit Corp., Seoul, Republic of Korea).

The prepared tooth was scanned using the intraoral scanner Medit i700 (Medit Corp., Seoul, Republic of Korea) (Figure 1) and the support die was digitally designed using Autodesk Fusion 360 (Autodesk Inc., San Francisco, CA, USA) design software [6,31,32,33,34]. The STL file was exported, and the tooth, together with its cylindrical support, was printed with an Asiga Max 3D printer (ASIGA Pty Ltd., Sydney, Australia), resulting in 20 specimens fabricated from ASIGA Model Resin (Asiga, Sydney, Australia) [32].

**Figure 2:**
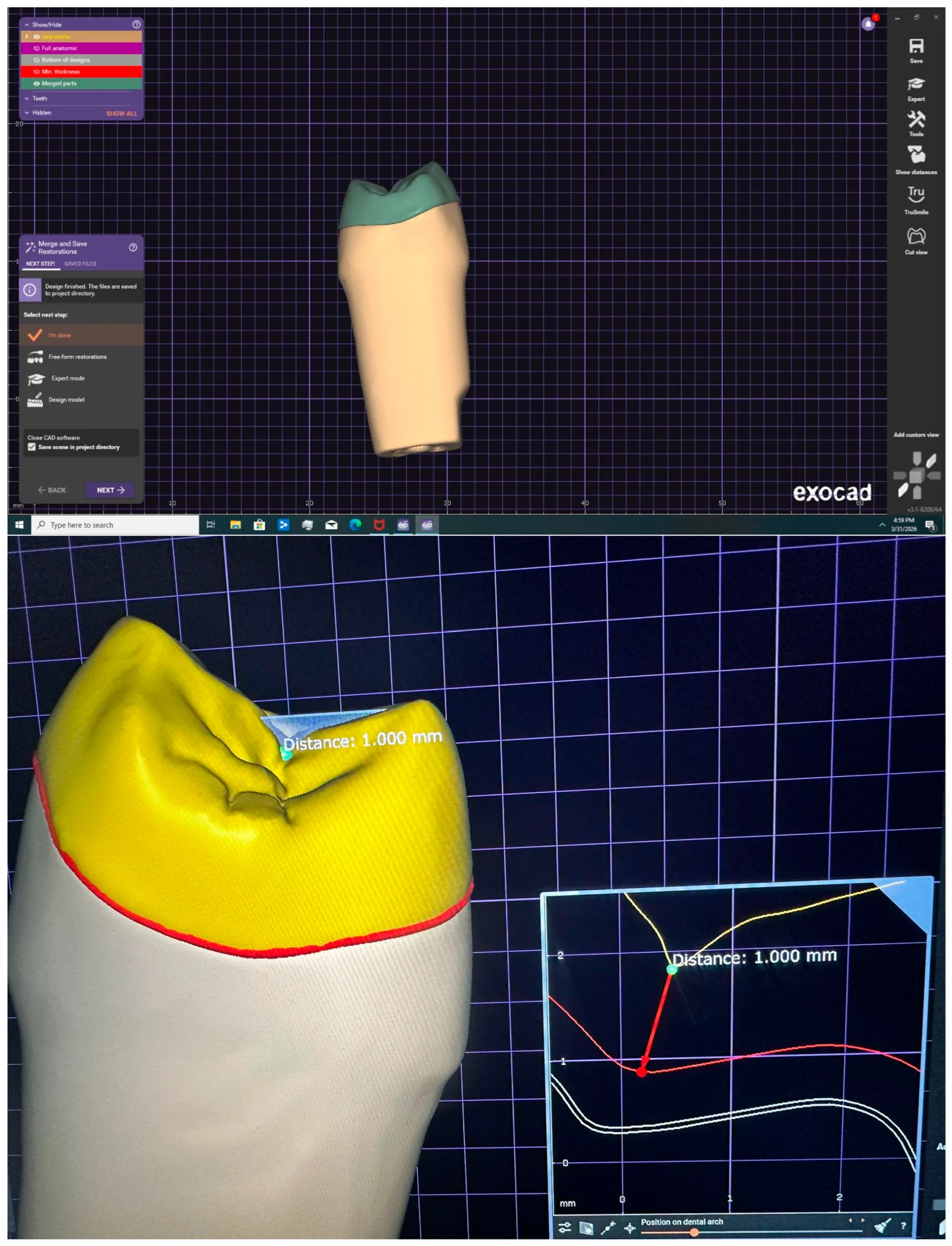

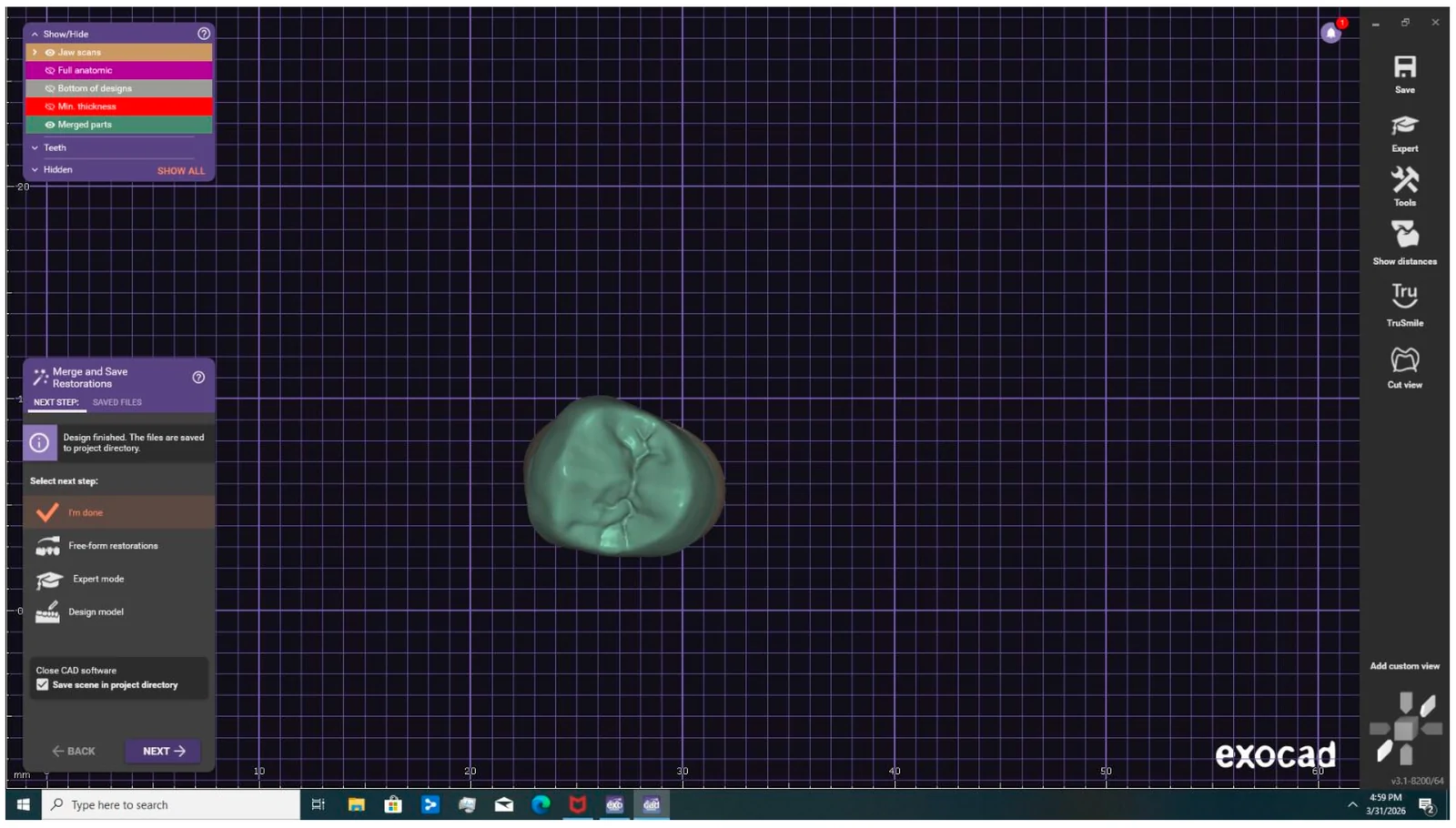
The occlusal veneer design in Exocad (Exocad GmbH, Darmstadt, Germany).

The occlusal veneer was also designed in the Exocad DentalDB 3.1 Rijeka designing software (Exocad GmbH, Darmstadt, Germany) (Figure 2), and printed using the same printer, at a build orientation angle of 0◦ and a layer thickness of 50 μm [33,35,36,37,38], resulting in 10 Samenco Crowntec occlusal veneers and 10 Voco V-Print occlusal veneers. The post-processing procedure included immersion in an ultrasonic bath containing 99% isopropyl alcohol for 3 min, air-drying, followed by photopolymerization for 10 min using a BB Midi Plus photopolymerization unit (MeccatroniCore, Pergine Valsugana, Italy).

**Figure 3:**
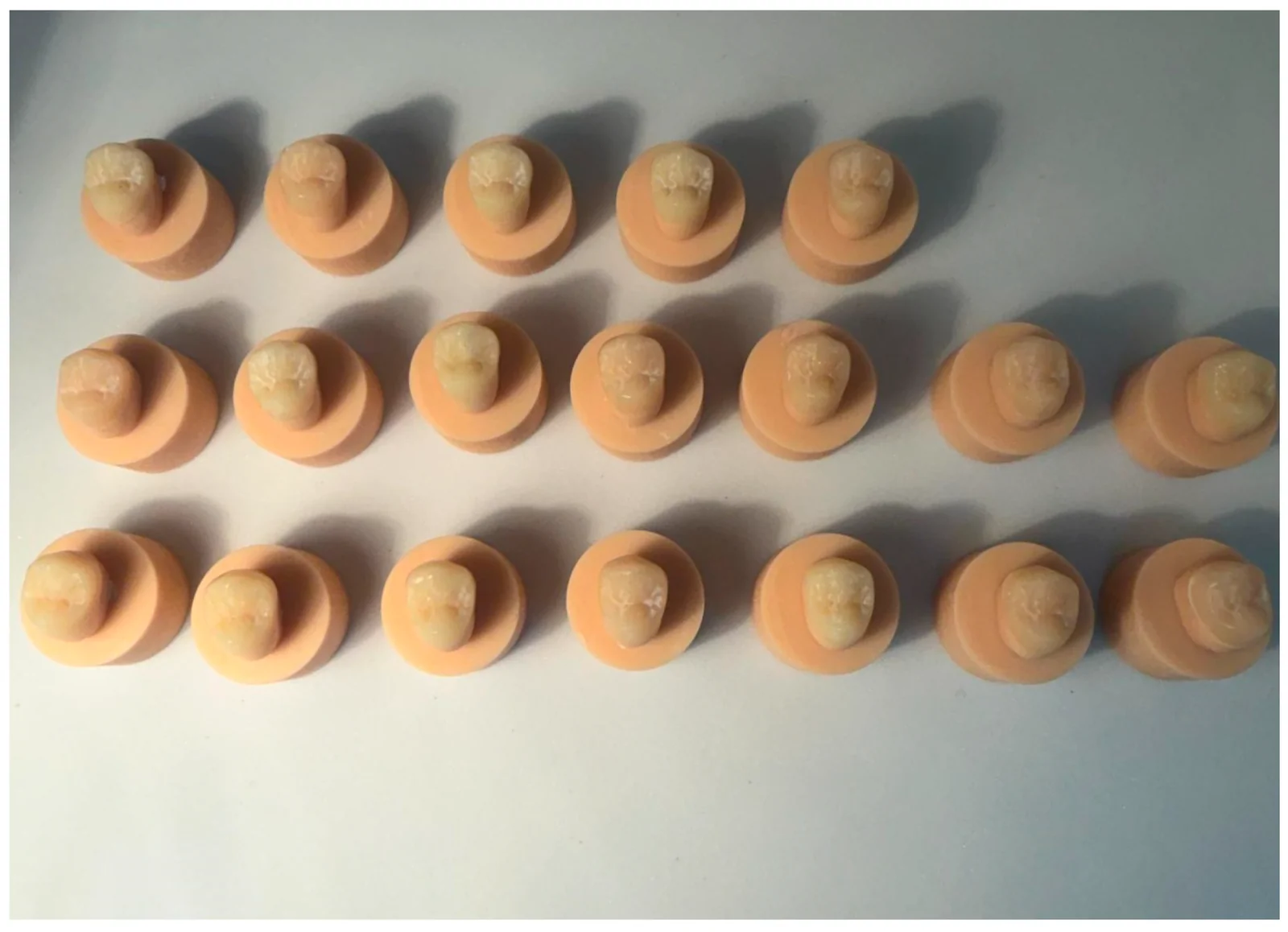
The printed resin abutments with Voco and Saremco occlusal veneers cemented.

The occlusal veneers were cemented with a constant pressure of approximately 20 N using Maxcem Elite Universal Resin Cement and photo-polymerized for 20 s on each side, according to the manufacturer’s instructions (Figure 3 [33]). Since no natural human tooth tissues were involved and resin dies were used instead, physiological adhesion could not be reproduced. Therefore, no further surface treatment was applied before the cementation process. Maxcem Elite, a self-adhesive dual-cure resin cement indicated for this type of indirect restoration, was chosen considering its dual-cure polymerization mechanism, which allows for adequate polymerization even in areas with limited light penetration [33].

To ensure the recommended mixing ratio and consistent handling of the material during the cementation procedure, the cement was poured directly from the automix syringe.

The samples were stored in distilled water before testing.

### 2.1. Fracture-Resistance Test

All cemented restorations on the resin dies were divided into two groups (*n* = 10) according to the restorative material used (Voco or Saremco) and were subsequently subjected to fracture-resistance testing using motorised test stand SAUTER TVM 5000N230N (KERN & SOHN GmbH, Balingen, Germany), with a maximum load capacity of 5 kN. The fracture-resistance test was performed under strictly standardized conditions.

**Figure 4:**
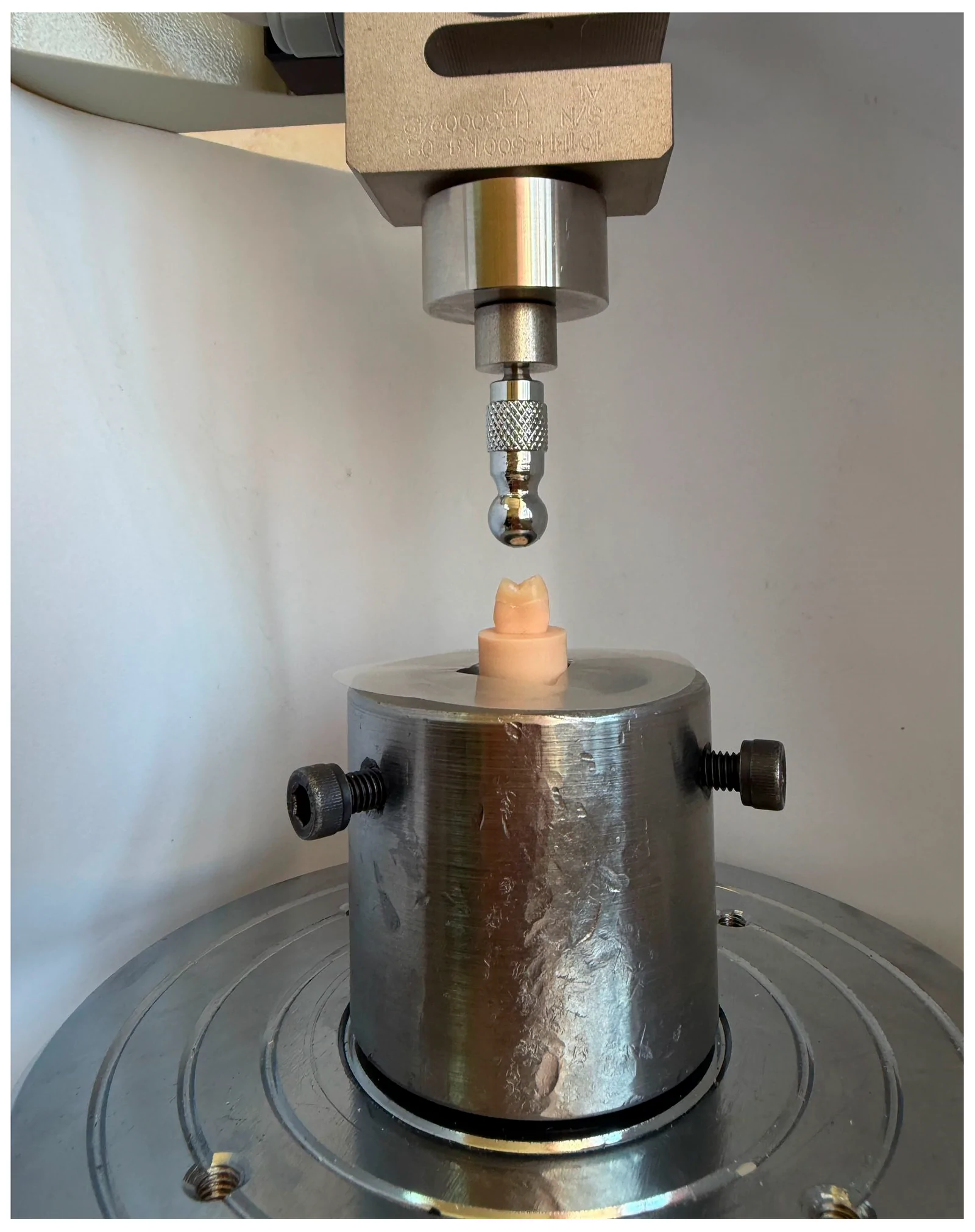
The compressive strength resistance mechanisms.

A 10 mm diameter stainless steel ball was perpendicularly applied (axial loading) at the center of the occlusal surface, at a crosshead speed of 5 mm/min, until failure of the occlusal veneer occurred (Figure 4). The use of a 10 mm diameter spherical indenter was selected as a standardized occlusal loading configuration. The choice is consistent with previously published fracture-resistance studies of indirect posterior restorations, including occlusal veneers [7,38,39,40,41,42,43].

All specimens were manually positioned by the same operator according to a controlled testing methodology, ensuring the alignment of the loading ball with the center of the occlusal surface before each test. The loading protocol was standardized by applying the force axially at the center of the occlusal surface, with identical crosshead speed, loading device, indenter, and specimen placement procedure for all specimens. Although small differences in positioning could not be totally excluded, the same experimental procedure was used for all specimens to reduce the variability and to allow the best possible comparison between the groups tested.

A 0.2 mm thick tin foil strip was placed between the ball and the restoration during loading, to standardize the contact surface and to uniformly distribute the applied forces. Comparable interposed tin foil layers have been used in previous mechanical tests of posterior dental restorations [33,38,39,40,41,42,43].

The force–time response was tracked using the dedicated AFH-LDcomputer software (Version 2.0.3.0; SAUTER, KERN & SOHN GmbH, Balingen, Germany). The fracture resistance was defined as the maximum load (peak force, N) recorded immediately before failure by the AFH-LD software The occurrence of fracture was additionally confirmed by the audible sound produced at the moment of specimen fracture and by visual examination, as a supplementary indication of the failure.

The absorbed energy (U) was calculated as the area under the load–displacement curve up to the fracture point, using numerical integration [44]:

$$U=\;\overset{m-1}{\underset{i=1}{\sum }}\frac{F_{i\;}+\;F_{i+1}}{2}\;\left(\Delta_{i+1}\;-\Delta_{i}\right)$$

where m is the total number of experimental data points, F_i_ represents the measured force (N), and Δ_i_ is the corresponding displacement (mm).

The fracture patterns were then classified according to the system proposed by Burke et al. (Table 2), allowing for standardized characterization of the failure mode across the experimental groups [45]. Burke’s classification has also been used in recent studies as a comprehensive and objective method for classifying fracture types, according to their characteristics and extent [6,7,41].

### 2.2. Fractographic Evaluation

Following the mechanical tests, to further characterize the fracture-related features associated with the identified failure patterns and to confirm the presence and degree of structural failure, representative fractured surfaces of the Voco and Saremco groups were chosen for scanning electron microscopy (SEM) analysis. The SEM specimens were selected to provide representative examples of each failure-code pattern identified in the study; consequently, all fracture codes observed in the experimental groups (specimens classified as catastrophic failure-Code 5, as well as representative specimens classified as Code 1 and Code 2) were illustrated by SEM images.

Accordingly, representative fractured surfaces of the Voco and Saremco occlusal veneers included in Code 5, Code 1, and Code 2 categories were analyzed using scanning electron microscopy (SEM) with a Vega Lmu scanning electron microscope (Tescan, Brno, Czech Republic) at magnifications of 30×, 50×, and 100×, and observation scales of 200 µm and 500 µm, in order to identify fracture-related features.

SEM analysis was performed as a qualitative fractographic assessment intended to complement the macroscopically identified failure modes (classified according to Burke’s classification) and to provide additional insight into the fracture characteristics.

All fractographic analyses as well as the failure mode classification were done by the same evaluator to reduce variability. The specimens allocated for fractographic analysis were selected based on their failure mode, comprising a representative sample of all the identified failure code patterns.

### 2.3. Statistical Analysis

Statistical analyses were performed using the JASP program (Version 0.95.4, JASP Team, Amsterdam, The Netherlands). With the help of this statistical program, the Shapiro–Wilk test was used to determine the normality of the data, and Levene’s test was used to determine the homogeneity of variances. An unpaired Student’s *t*-test was used in order to identify statistically significant differences between the recorded forces corresponding to the two materials. No experimental results were excluded from the analysis.

### 2.4. Weibull Analysis

Weibull analysis was conducted using OriginPro 2026 SR1, version 10.3.0.197 (OriginLab Corporation, Northampton, MA, USA) to determine the Weibull modulus (m) and the characteristic fracture strength (σ_0_), with 95% confidence intervals (CI).

The Weibull analysis summarizes the cumulative probability of failure (Pf), including two important parameters: the Weibull modulus (m), which describes the shape parameter, and the characteristic strength (σ_0_), which represents the scale parameter. This relationship is expressed by the following formula [46]:

Pf = 1 − exp[−(σ/σ_0_)^m^]

The Weibull modulus (m) reflects the reliability of a material, with higher values indicating more uniform results and lower values indicating greater variability. The characteristic strength (σ_0_) corresponds to the load associated with a 63.2% probability of failure [13].

The Weibull modulus is an important parameter that may change depending on the filler proportion, particle size, and the mechanical testing method performed [47].

## 3. Results

### 3.1. Statistical Analysis Results

The power analysis was performed using G*Power v3.1 (HHU Düsseldorf) with α = 0.05 and power = 0.80, confirming that a sample size of 10 specimens per group was sufficient for detecting meaningful statistical differences. The Shapiro–Wilk test and Levene’s test indicated normal data distribution (*p* > 0.05) and homogeneity of variances (*p* > 0.05), respectively. Consequently, an independent Student’s *t*-test was applied, revealing no statistically significant differences in force values between the Voco and Saremco samples (*p* = 0.146).

### 3.2. Failure Force Values

**Figure 5:**
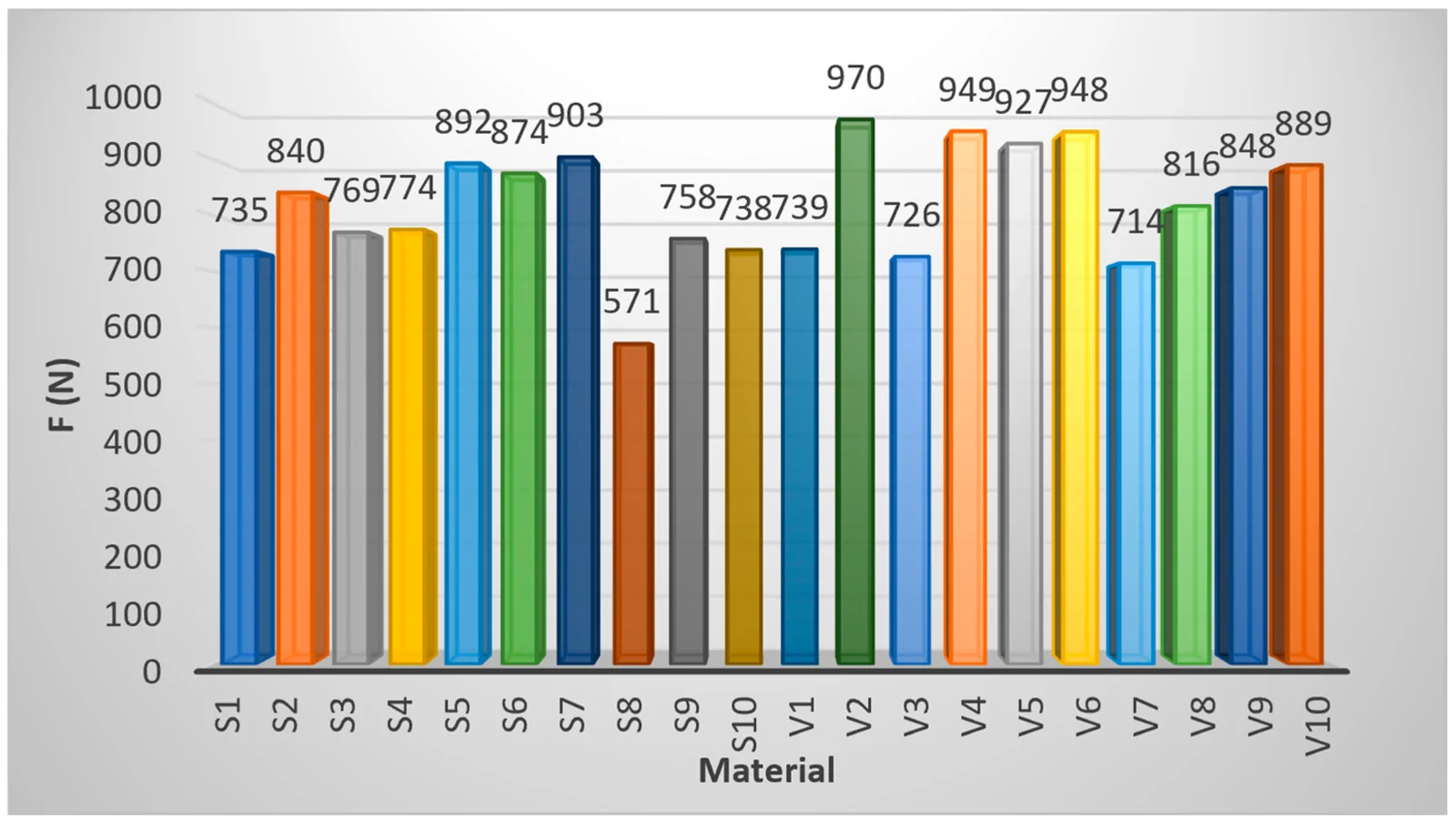
Means of F (N) for Voco and Saremco.

The maximum forces (N) recorded at the fracture point are presented in Figure 5 and Table 3. The Voco group showed higher mean force values compared with the Saremco group (Voco—852.6 N vs. Saremco—785.4 N). The highest force value was recorded for V2 (970 N), whereas the lowest value was observed for S8 (571 N).

### 3.3. Load–Displacement Curve

Displacement was not directly recorded by the testing machine and was calculated from the time elapsed after initial contact with the specimen using the relation d=vt.

**Figure 6:**
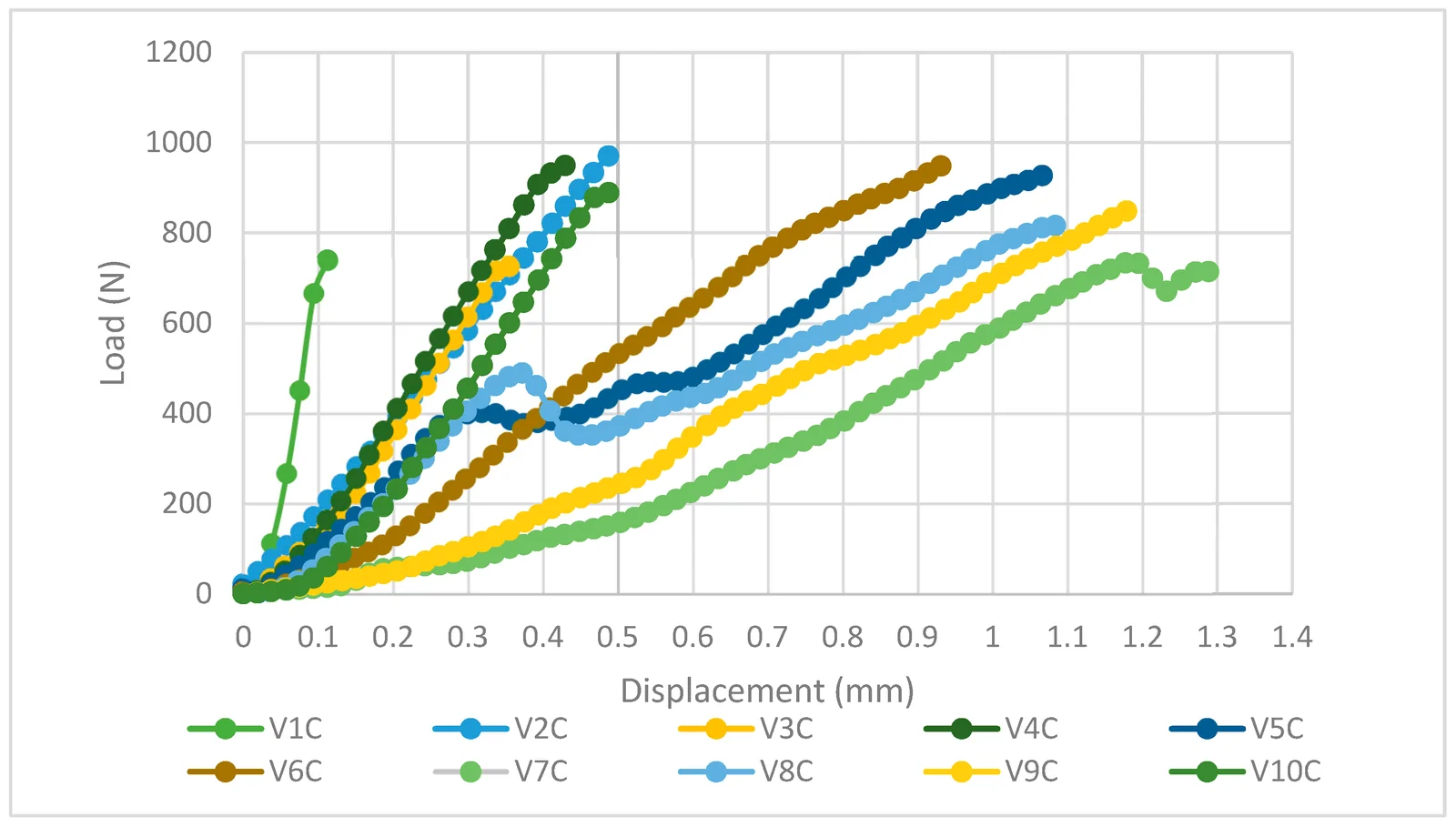
Representative load (N)–displacement (mm) curve of Voco occlusal veneers.

The load–displacement curve representation for the Voco material is shown in Figure 6. The load–displacement curves indicated a gradual increase in load with displacement until the failure point. The fracture loads obtained ranged from 714 to 970 N, and the corresponding displacements ranged from 0.11 to 1.28 mm, showing some variability in the mechanical behavior of the samples tested. However, despite this variation, the load–displacement response showed a consistent increase in load until the point of fracture.

**Figure 7:**
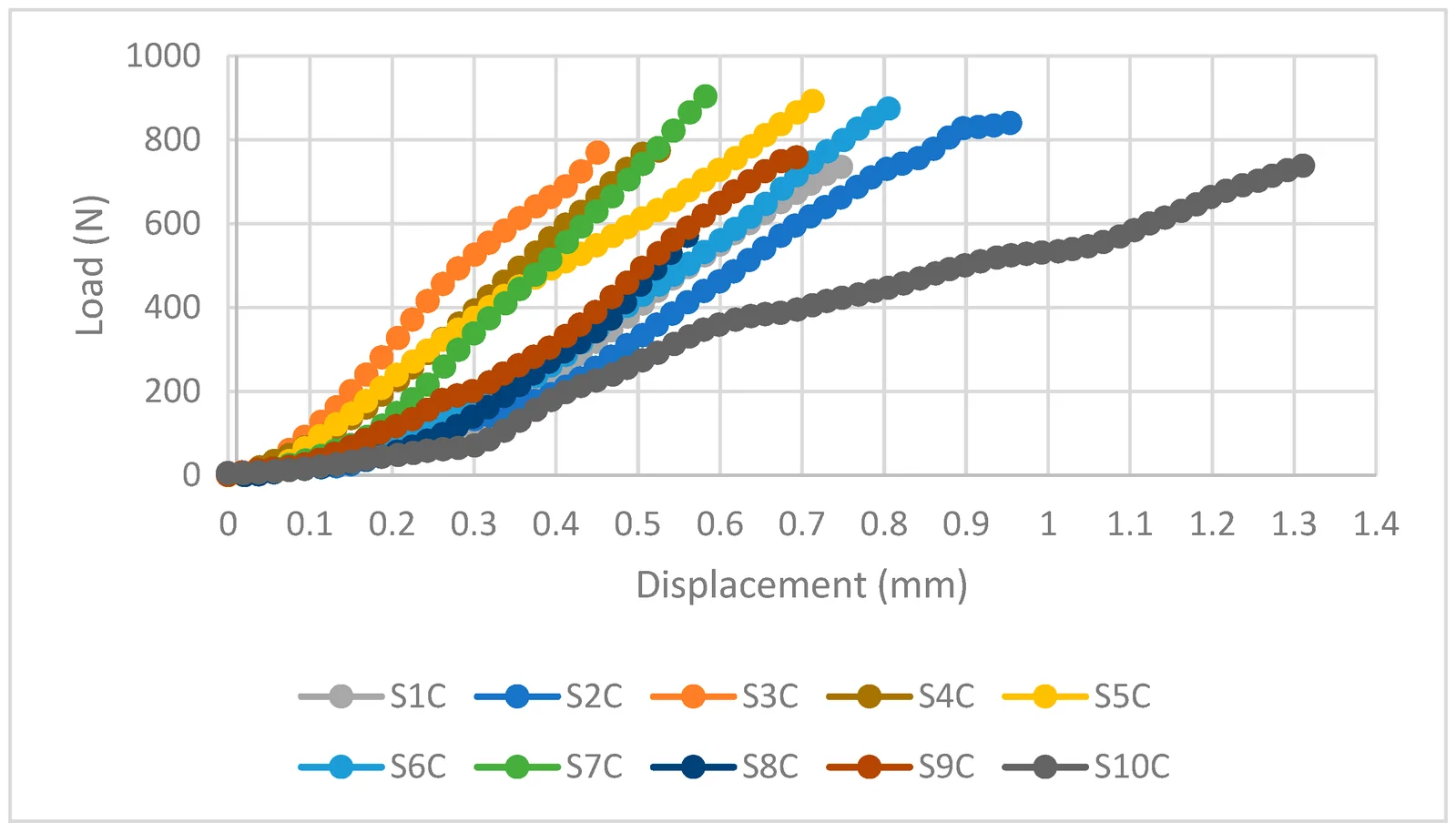
Representative load (N)–displacement (mm/s) curve of Saremco occlusal veneers.

The load–displacement curve corresponding to the Saremco material is presented in Figure 7. The force values ranged from 571 to 903 N, while the displacement values ranged from 0.45 to 1.31 mm. In comparison with Voco, which showed fracture loads ranging from 714 to 970 N and displacement values from 0.11 to 1.28 mm, Saremco had a wider range of displacement values and lower fracture-load values overall.

Consequently, Saremco samples showed higher deflection (ranging between 0.45 and 1.31 mm) compared with the Voco samples (ranging between 0.11 and 1.28 mm). The lower fracture-load range recorded for Saremco may indicate a lower load-bearing capacity under the force conditions applied in the present study. However, the similar ranges of displacements at failure suggest that the difference in fracture resistance was not necessarily associated with a markedly different displacement response at failure.

### 3.4. Fracture Strength Test- Energy at Failure

**Figure 8:**
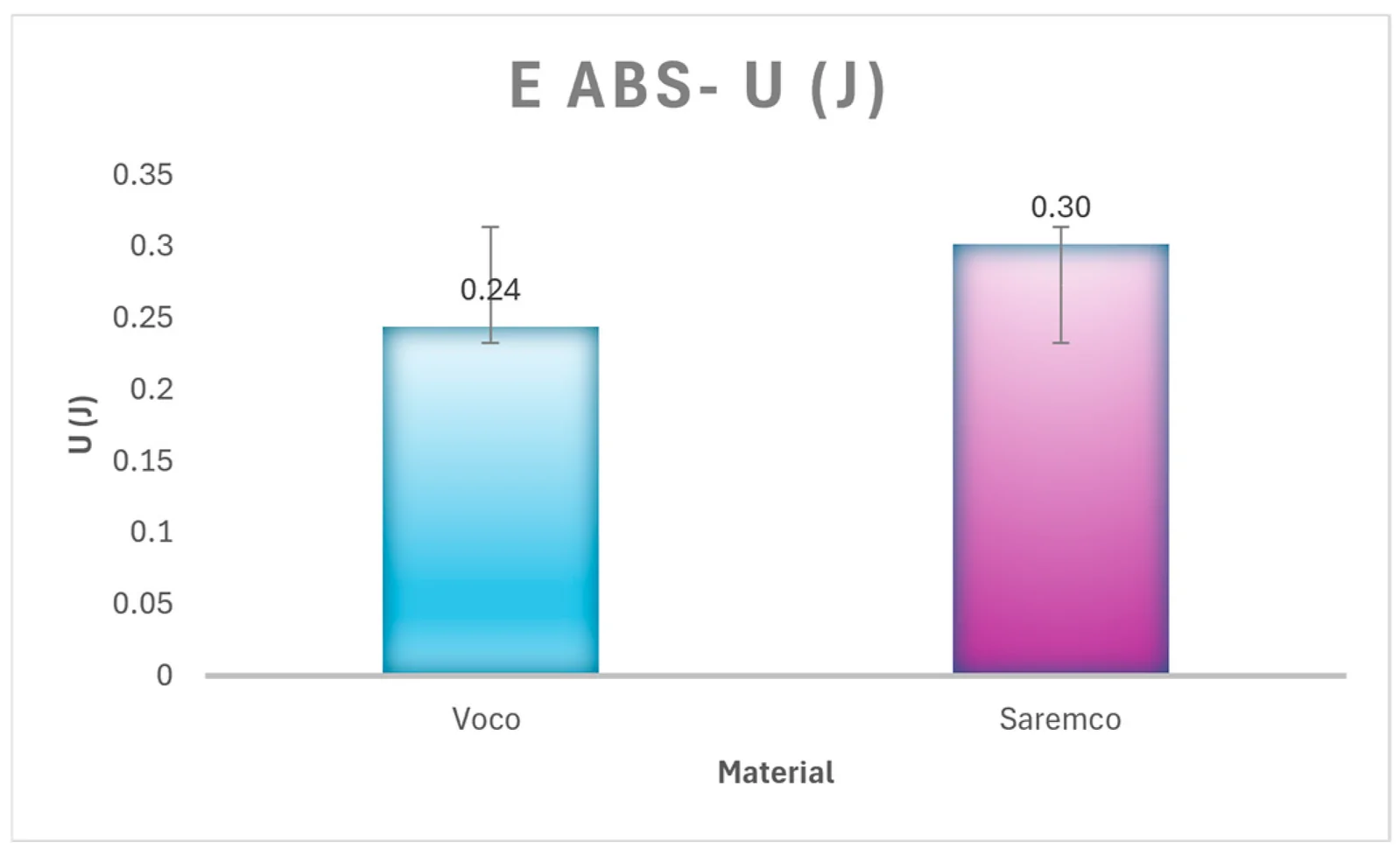
The mean absorbed energy at failure (J) and standard deviation.

The mean energy at failure for Voco was 0.301 J (with an average force of 852.6 N and a mean strain of 742.10 µm), and for Saremco was 0.244 J (with an average force of 785.4 N and an average strain of 734.23 µm) (Figure 8).

### 3.5. Fracture Pattern Classification

The fracture pattern for each specimen, in accordance with Burke’s classification [47], is represented in Table 4.

It can be concluded that Voco occlusal veneers exhibited more catastrophic failure in comparison to Saremco occlusal veneers. Voco specimens fractured into a greater number of fragments, suggesting a more pronounced failure pattern than that observed in the Saremco group.

### 3.6. Weibull Analysis Results

**Figure 9:**
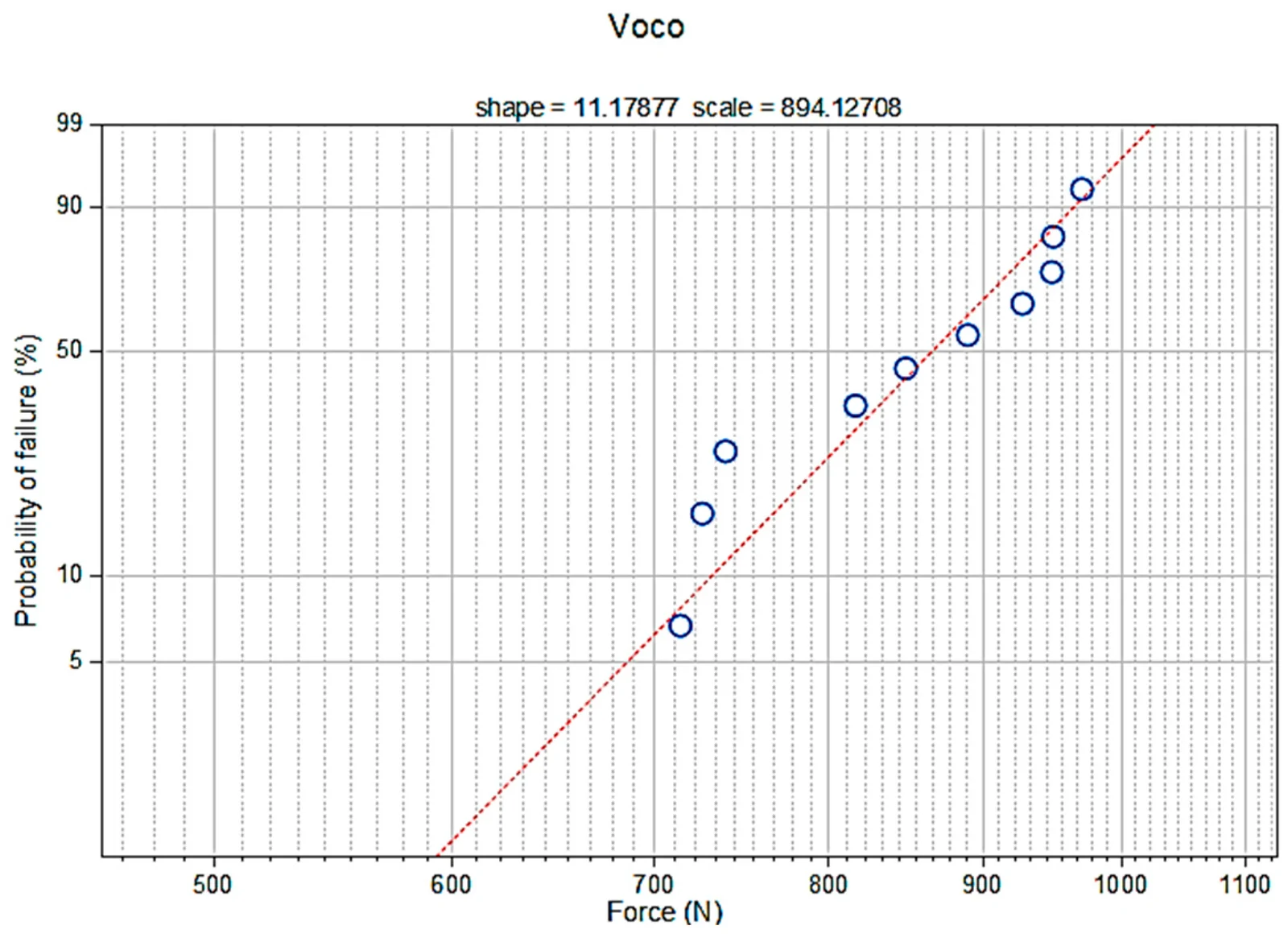
Weibull probability plot of fracture load values for the Voco group samples (n = 10).

**Figure 10:**
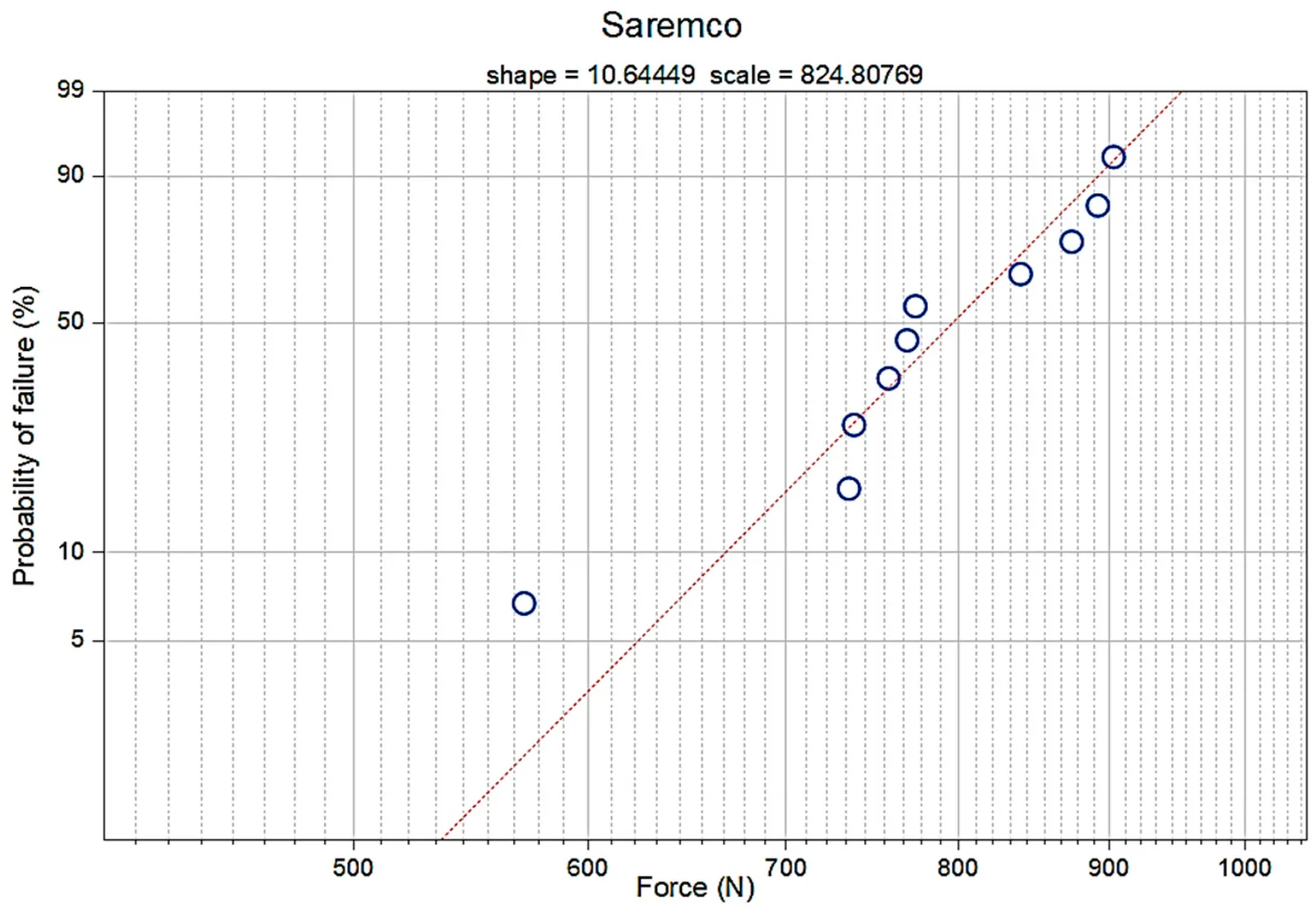
Weibull probability plot of fracture load values for the Saremco group samples (n = 10).

The Weibull analysis revealed a Weibull modulus for Voco of 11.178 and a characteristic strength of 894.127, in comparison with Saremco, which recorded a Weibull modulus of 10.644 and a characteristic strength of 824.807 (Table 5). The Weibull probability plots for both materials are presented in Figure 9 and Figure 10.

### 3.7. Fractography Evaluation

The SEM fractographic evaluation revealed significant fracture features associated with crack initiation and propagation.

**Figure 11:**
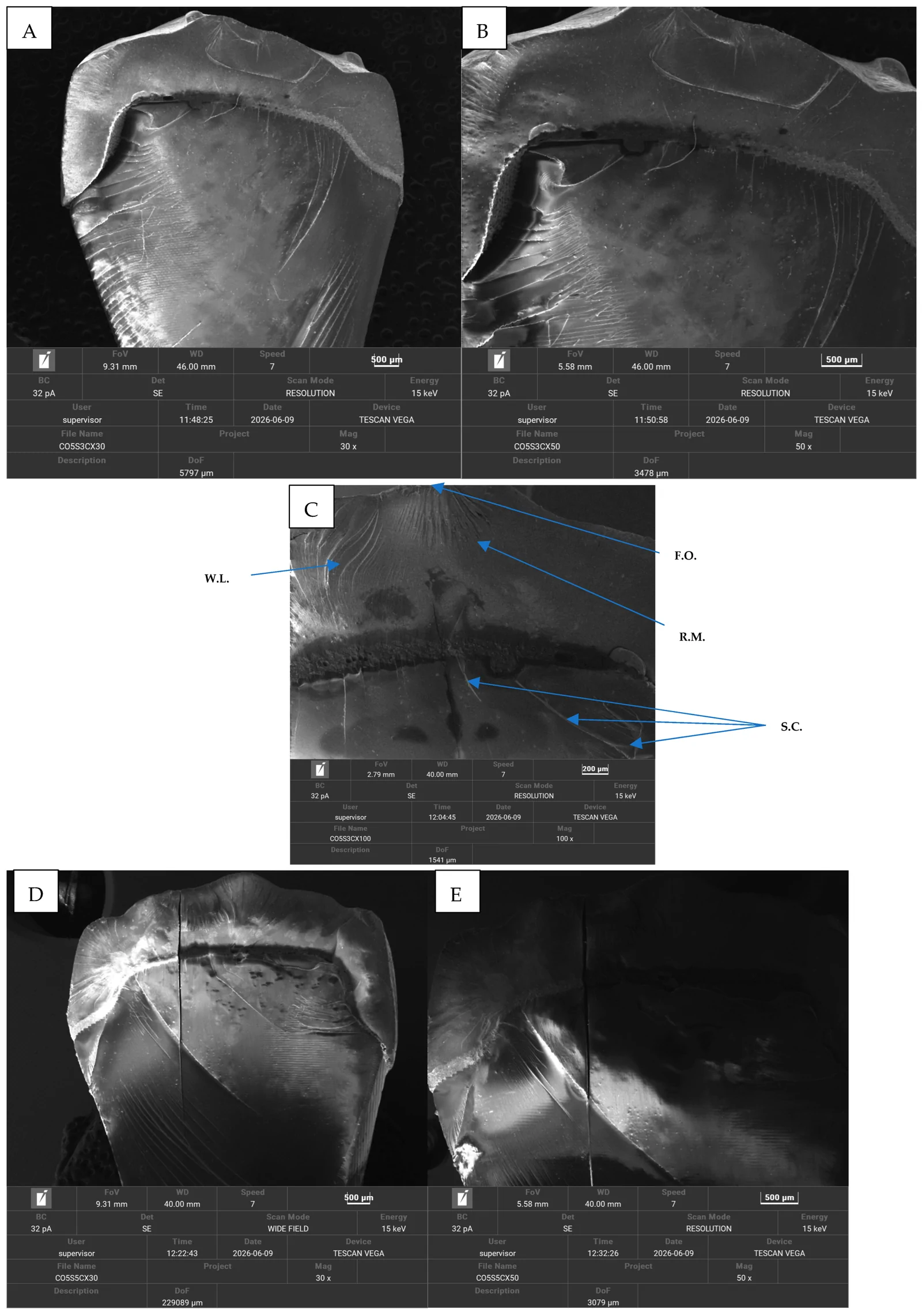

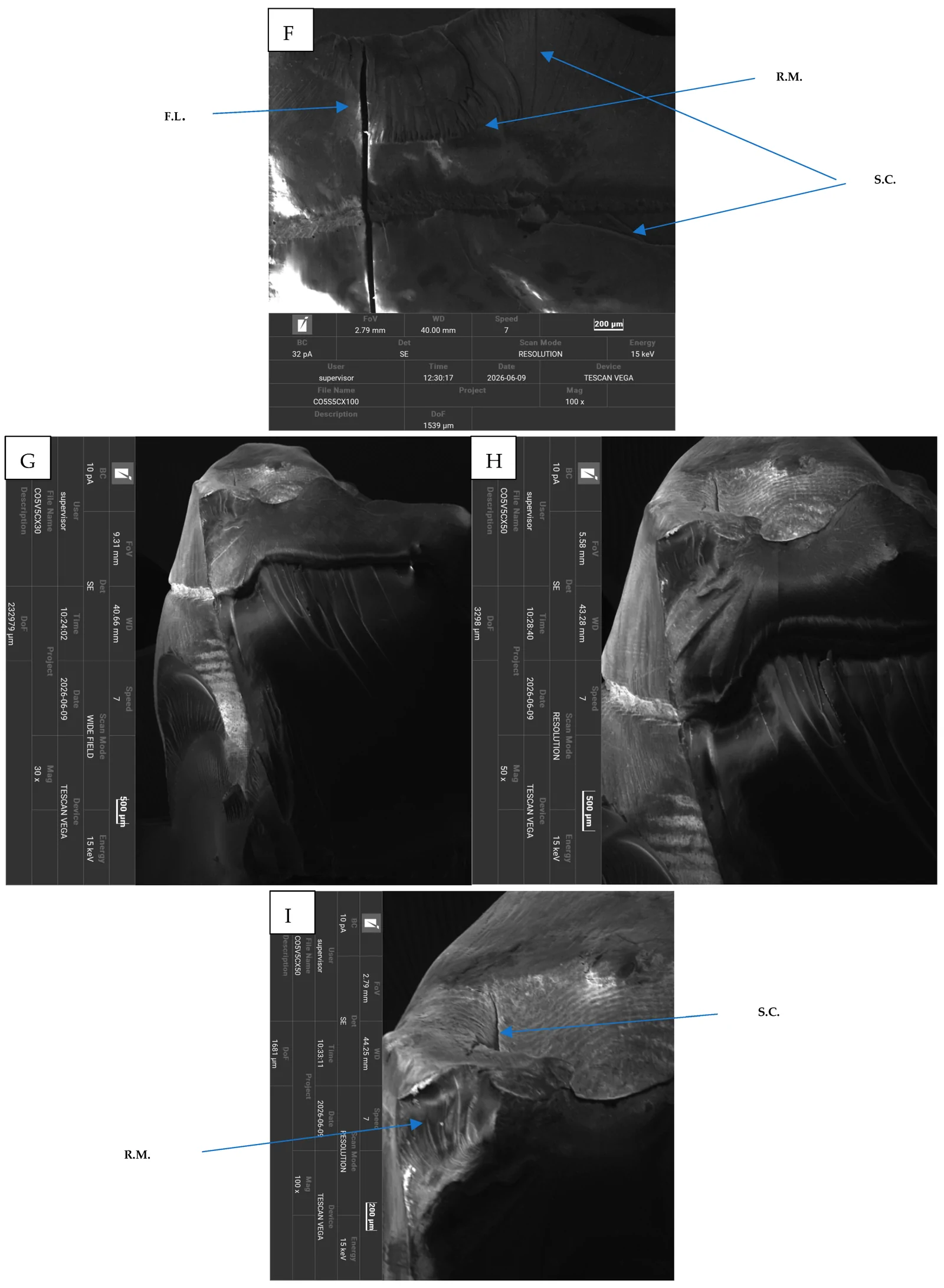

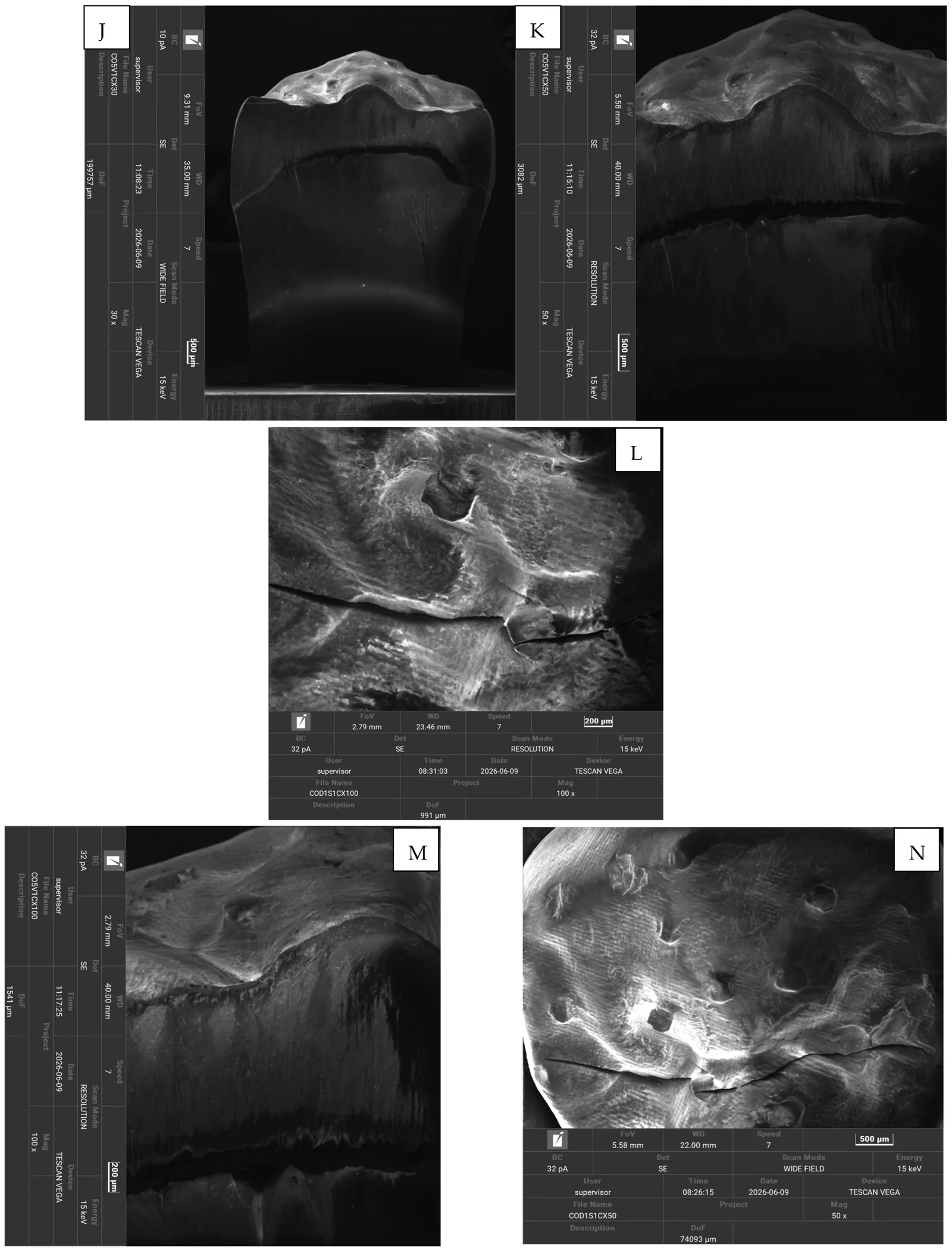

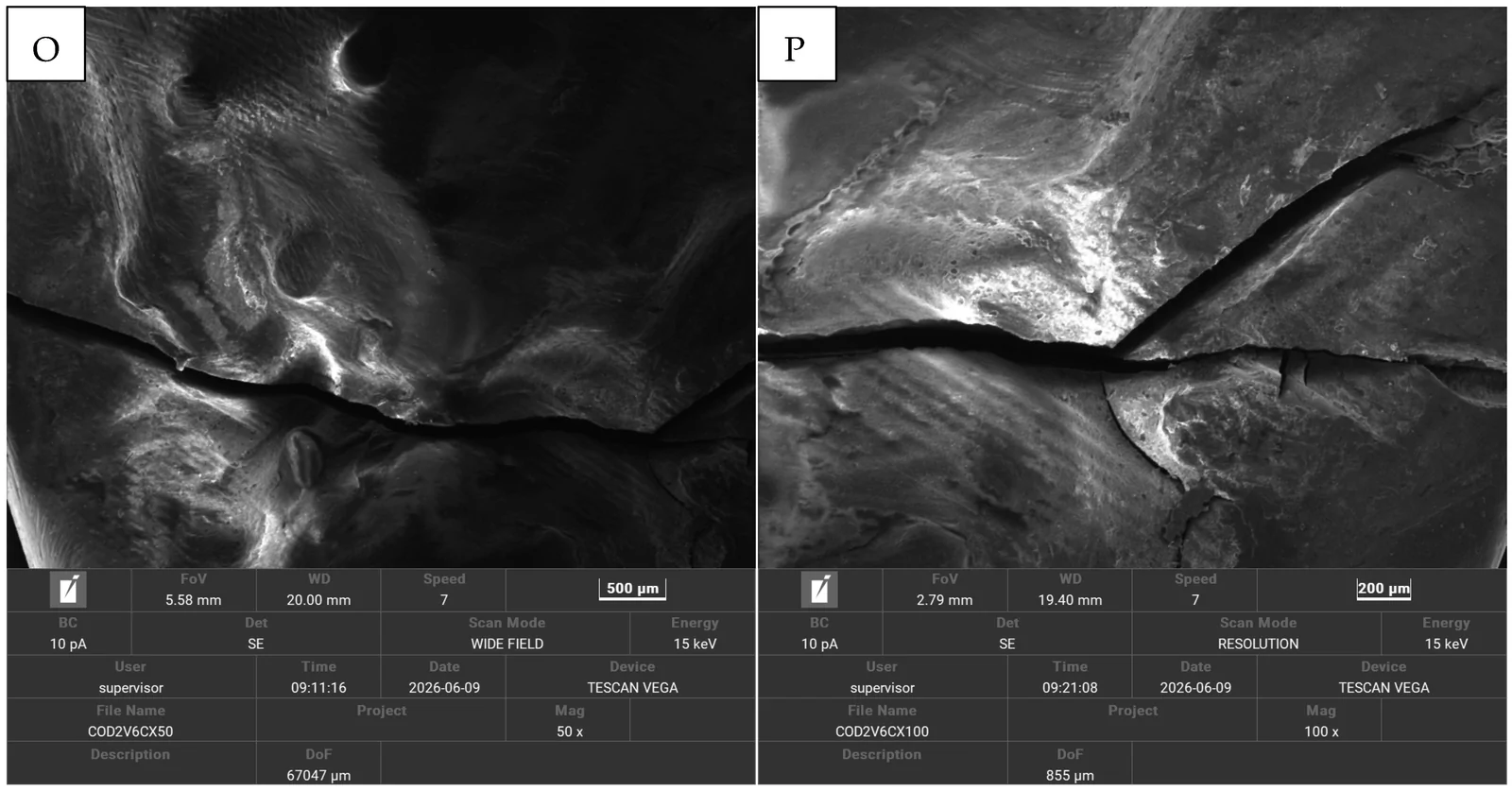
The fractographic examination at different magnifications and scales of observation for Saremco 1-Code 5 (**A**–**C**), Saremco 2-Code 5 (**D**–**F**), Voco 1-Code 5 (**G**–**I**), Voco 2-Code 5 (**J**–**L**), Saremco 3-Code 1 (**M**,**N**), Voco 3-Code 2 (**O**,**P**); F.L.—fracture line; W.L.—Wallner line; F.O.—fracture origin; R.M.—river markings; S.C.—secondary cracks.

The first Saremco sample showed numerous fracture features: Wallner’s lines and river markings, which appear to converge toward probable fracture origin regions and are associated with secondary cracks (Figure 11A–C).

Figure 11D,E highlights a pronounced fracture line, which may correspond to the main fracture trajectory generated during compressive loading.

The SEM representation of the Voco sample (Figure 11I) identified fracture-related characteristics, but these were less pronounced in comparison with the Saremco samples. The fracture surfaces generally demonstrated a more homogeneous appearance, with fewer evident fracture-related features.

Figure 11M,N corresponding to a Code 1 Saremco sample, presents the occlusal surface with the midway fracture line. Also, in the case of Voco Code 2 (Figure 11O,P), the occlusal examination showed a bifurcation of the principal fracture trajectory. This branching pattern may indicate local variations in crack propagation during failure.

## 4. Discussion

In the literature, occlusal veneers are regarded as a modern and conservative solution for the management of severe erosive lesions [48]. Erosion and abrasion are common clinical findings associated with extensive loss of tooth structure reported in previous studies [30]. However, the optimal restorative material has not been determined yet, according to researchers [49]. Numerous restorative materials, dental tissue preparation techniques and substrate preparation protocols are still under investigation. The literature reports a wide range of materials studied for occlusal veneers (such as zirconia, resin nano-ceramics, lithium disilicate and feldspathic ceramics) [50,51]. Among these materials, zirconia has shown promising results in terms of overall stability, but its major disadvantage is its demanding cementation procedure, which can ultimately lead to debonding [21]. A study that investigated the fatigue resistance of posterior occlusal veneers made of ceramic and composite resin found that resin composites demonstrated better outcomes than glass–ceramics, probably due to their elastic modulus being closer to that of dentin, which constitutes a great advantage [52]. Nevertheless, in the current state of knowledge, studies on newly developing 3D-printed materials for this application are still limited. This study was designed to address this gap by investigating these materials, aiming to provide clinicians with new insights.

It has been noted that the preparation design and the type of dental substrate treatment procedure can influence the durability and marginal adaptability of occlusal veneers [53]. For example, a study examining the impact of preparation design on stress distribution and fracture resistance found that an increase in the number of axial walls led to higher stress concentrations within the restoration and a reduction in fracture resistance [54].

In addition, comparisons between various veneers and traditional crowns revealed that conservative restorations performed better than conventional full crowns, highlighting the good performance of minimally invasive approaches [55].

Also, according to some studies, the mechanical performance of 3D-manufactured composite materials is more and more similar to that of conventional materials [19]. The overall good mechanical properties were also demonstrated in this study, with force values capable of withstanding masticatory forces, which are known to range between 597 N and 847 N (molar area for both sexes), as reported in one study [56]. Values from this study ranged from 970 N (recorded by V2) to 571 N (recorded by S8), which are similar to those reported in other research. The first null hypothesis was accepted, as no statistically significant differences in fracture resistance were observed between the two materials.

However, when comparing PICN material restorations (Vita Enamic, 0.3 mm thickness) with lithium disilicate, Vita Enamic showed a fracture load ranging from 547 to 846 N; these values are above normal occlusal loading (50–300 N), yet below the maximum bite force approximated at 1200 N [57]. On the other hand, in a comparison between a 3D printing material and a PICN material, the 3D-printed material demonstrated inferior mechanical properties [58].

Taking into account the occlusal veneer thickness parameter, another study reported that 0.7–1.0 mm thick glass–ceramic occlusal veneers demonstrated fracture-resistance values applicable in the posterior area that exceeded the minimum required limit, which was stated to be between 500 and 700 N [54]. However, it was highlighted that only 37–40% of this force is used during mastication [59,60].

Regarding the mean energy at failure in this study, the values ranged between 0.301 J (for Voco) and 0.244 J (for Saremco). These values can be compared with the absorbed energy values reported in another study, where absorbed energies of 0.077–0.231 J were measured for ceramic materials. That study reported that an absorbed energy of 0.231 J was required to produce splitting of the tested crown, while lower energy levels resulted only in delamination [31].

The fractographic analysis revealed different fracture patterns and fracture features, a finding that was also supported by the macroscopic Burke fracture pattern classification. Therefore, the second null hypothesis was rejected. The lower-filled 3D resin demonstrated a more uniform fractographic appearance than the highly filled material, an observation that was also supported by its higher absorbed energy, suggesting greater toughness. However, according to Burke’s fracture classification, the lower-filled material presented higher catastrophic failures.

The findings related to the Weibull analysis indicate that Voco demonstrated both a higher fracture resistance (its higher characteristic strength) and a more reliable mechanical behavior than Saremco (determined by its higher Weibull modulus). Consequently, the third null hypothesis was rejected, as there were differences in the Weibull probability of failure between the two 3D-printed resin materials. The relatively limited sample size (n = 10) represents a limitation of the Weibull analysis, and the resulting Weibull modulus values should therefore be interpreted cautiously.

The inferior mechanical properties of 3D-printed materials, as reported in different research papers, can be attributed to structural defects resulting from the printing process, such as voids or areas of incomplete polymerization, which may act as stress points and cause fractures under applied loads [28]. However, according to a review comparing the mechanical performance of 3D-printed provisional restorations with those fabricated using subtractive and conventional manufacturing methods, 3D printing demonstrated superior mechanical performance [61]. Nonetheless, the comparison of fracture resistance between additive and subtractive materials, or among other 3D materials, remains insufficiently addressed in the literature. Research in these areas is still ongoing.

Another important factor influencing the mechanical performance is the elastic modulus of the luting cement, which determines the way in which stresses are transferred within the restoration, affecting its fracture resistance [21]. The chosen cement, Maxcem Elite, has an elastic modulus of 4 GPa and can be considered a cement with a compatible elastic modulus, which may contribute to a more favorable distribution and transmission of masticatory forces [22]. An adhesive resin cementation was also chosen because restorations made of ceramic studied in previous studies have been shown to be more fracture-resistant than those cemented with conventional cements [29].

Before being used in a clinical setting, the fracture resistance of restorative materials must be evaluated in vitro to establish their suitability as a therapeutic option [62]. Moreover, since fracture resistance is strongly related to the long-term performance of dental restorations, as reported in previous studies, this test was selected for evaluation in the present study [62]. Nevertheless, the forces acting within the oral environment are not fully compressive, as observed in fracture tests, because oral components are exposed to a combination of different forces [63]. Consequently, the results should be interpreted with caution in a clinical context, as the experimental conditions were limited to static axial loading.

In the present study, two 3D-printed resin-based materials with different intended clinical applications, as mentioned by the manufacturers, were compared: a provisional resin (Voco V-Print C&B Temp) and a definitive resin (Saremco print Crowntec). The selected materials represent two clinically relevant categories of 3D-printable restorative resins currently available for provisional and definitive applications. For the purpose of the present study, the investigated materials were grouped according to their reported inorganic filler content. Saremco print Crowntec was considered a medium-to-high filled material (30–50% inorganic filler), whereas Voco was classified as a lower-filled material. Both materials are manufactured using the same additive manufacturing technology but differ in internal composition, inorganic filler content, and intended clinical use. The comparison of these materials gives insight into the influence of the material formulation on the mechanical performance of additively manufactured restorations. Such information may assist clinicians in selecting the appropriate material according to the clinical scenario.

The limitations of this study include the use of resin dies, which cannot fully reproduce the biomechanical properties of natural dental tissues. Therefore, the supporting substrate may influence the fracture resistance and fracture patterns.

Also, another limitation of this study is its in vitro nature and the lack of correlation with a degradation method. The present static load-to-fracture test provides a standardized method to measure the fracture resistance of restorations under controlled laboratory conditions, but it does not completely simulate the complex intraoral conditions, which include cyclic fatigue, temperature changes, and variations in occlusal contacts.

Thus, the results represent a direct comparison of the mechanical behaviors of the two 3D-printed materials, which are currently of considerable interest and remain the subject of ongoing research, under the standardized in vitro conditions used in this study.

This study offers a broader assessment of the mechanical performance of the two materials under standardized experimental conditions, complemented by a standardized macroscopic failure classification according to Burke’s criteria and a microscopic assessment using SEM. Moreover, it provides another perspective on failure prediction and data reliability through the inclusion of Weibull analysis. Consequently, these complementary assessments provide a more complete picture of the mechanical performance and failure behaviors of the two investigated materials.

## 5. Conclusions

1. There were no statistically significant differences in the mean force values between the highly filled and low-filled 3D resin composites.

The mean energy at failure was 0.301 J for the low-filled resin composite, while the highly filled resin composite recorded an absorbed energy (E_abs_) of 0.244 J, indicating similar fracture behavior between the two materials. A slightly higher mean E_abs_ value, recorded for the lower-filled material, suggests greater toughness.

2. According to Burke’s failure classification, the low-filled material exhibited a more catastrophic fracture pattern compared with the highly filled material.
3. The fractographic analysis revealed that the highly filled material samples had more pronounced fracture features and complex crack propagation, while the low-filled material showed smoother fracture surfaces with fewer features and more uniform failure patterns.
4. The Weibull analysis revealed greater predictability and higher fracture strength for the lower-filled material, recording a Weibull modulus of 11.178 and a characteristic strength of 894.127.

## Figures and Tables

**Table 1:** Materials—manufacturer and composition.

Material	Manufacturer	Composition
Voco V-print C&B Temp A2	VOCO GmbH, Cuxhaven, Germany	Ceramic-filled (26 wt% inorganic fillers) hybrid material with aliphatic urethane dimethacrylate (>10–25%), aliphatic acrylate (>2.5–10%), triethylene glycol dimethacrylate (>2.5–10%), and diphenyl(2,4,6-trimethylbenzoyl) phosphine oxide (max. 2.5%).
Saremco Print Crowntec A2	Saremco Dental AG, Rebstein, Switzerland	Esterification products of 4,4′-isopropylidiphenol, ethoxylated and 2-methylprop-2enoic acid, silanized dental glass, pyrogenic silica, initiators. Total content of inorganic fillers (particle size 0.7 μm) is 30–50% by mass.
Maxcem Elite Clear	SDS Kerr, Orange, CA, USA	Glyceroldimethacrylate dihydrogen phosphate (GPDM) hydroxyethyl methacrylate (HEMA), 4-methoxyphenol (MEHQ), cumolhydroperoxide (CHPO), methacrylate ester monomers, titanium dioxide, and pigments.

**Table 2:** Failure modes according to Burk’s classification.

Code	Interpretation
1	Minor crack or crack in the occlusal veneer.
2	Less than 50% of the occlusal veneer is detached.
3	Fracture along the midline, with half of the occlusal veneer displaced or lost.
4	More than 50% of the occlusal veneer is lost.
5	Extensive fracture of the resin die and/or the occlusal veneer.

**Table 3:** Descriptive statistics—mean, SD.

	N	Missing	Mean	Standard Deviation	SE of Mean	Lower 95% CI of Mean	Upper 95% CI of Mean
Saremco	10	0	785.4	98.84241	31.25671	714.6924	856.1076
Voco	10	0	852.6	99.1858	31.3653	781.64676	923.55324

**Table 4:** Fracture codes in accordance with Burke’s classification.

Material	Code 1	Code 2	Code 3	Code 4	Code 5
Voco	0	1	0	0	9
Saremco	3	0	0	0	7

**Table 5:** Weibull analysis for the fracture strength of Saremco and Voco samples; m—Weibull shape, Weibull modulus; σ—characteristic strength (N), Weibull scale; SD—standard deviation.

Material	Distribution	Parameter	Estimate	Lower 95%	Upper 95%	SD of Estimate
Saremco	Weibull	Scale alpha (σ_0_)	824.80769	775.87771	876.82339	25.73588
		Shape beta(m)	10.64449	6.44075	17.59192	2.7285
Voco	Weibull	Scale alpha (σ_0_)	894.12708	843.41771	947.88528	26.63524
		Shape beta (m)	11.17877	6.68797	18.685	2.92995

## Data Availability

The original contributions presented in this study are included in the article. Further inquiries can be directed to the corresponding author.
